# A Nutrition Intervention to Promote the Consumption of Pulse-Based Foods in Childcare Centers: Protocol for a Multimethod Study

**DOI:** 10.2196/22775

**Published:** 2020-12-24

**Authors:** Hiwot Abebe Haileslassie, Renee Ramikie, Hassan Vatanparast, D Dan Ramdath, Amanda Froehlich Chow, Phyllis Shand, Rachel Engler-Stringer, Jessica RL Lieffers, Shannon Hood-Niefer, Carol Henry

**Affiliations:** 1 College of Pharmacy and Nutrition University of Saskatchewan Saskatoon, SK Canada; 2 Department of Applied Human Sciences University of Prince Edward Island Charlottetown, PE Canada; 3 School of Public Health University of Saskatchewan Saskatoon, SK Canada; 4 College of Agriculture and Bioresources University of Saskatchewan Saskatoon, SK Canada; 5 Department of Community Health and Epidemiology University of Saskatchewan Saskatoon, SK Canada; 6 Saskatchewan Food Industry Development Centre Inc Saskatoon, SK Canada

**Keywords:** behavior change, childcare center, intervention mapping, nutrition intervention, preschool children, pulse

## Abstract

**Background:**

Plant-based foods, including pulses (dry beans, lentils, chickpeas, and peas), have gained worldwide attention owing to their health and environmental benefits. Despite high production, the consumption of pulses is low in Canada. Behavior change interventions systematically designed to promote the consumption of pulse-based foods are scarce.

**Objective:**

We describe the utilization of intervention mapping (IM) in the development of a multicomponent nutrition intervention aimed at promoting consumption of pulse-based foods among preschool children in childcare centers in Saskatchewan, Canada.

**Methods:**

The Pulse Discovery Toolkit intervention was developed following the six steps of the IM protocol. Decisions at each step were either based upon literature review, expert consultation, pretesting, or a combination of these. Following the initial phase of the study, which focused on intervention development, phases II and III of the study were concerned with pilot testing and roll-out of the intervention, respectively. In total, one, two, and four childcare centers participated in phases I, II, and III, respectively. A multimethod approach was designed to evaluate the intervention during pilot testing and roll-out.

**Results:**

The application of IM steps 1 to 3 in phase I resulted in the creation of performance objectives at different levels, including at the individual level (preschool children), and the social and environmental levels (parents, early childhood educators, and cooks). These objectives were then used to create a matrix of objectives matching the constructs of the social cognitive theory while taking Piaget cognitive development into consideration. This step was followed by defining program components, implementation, adoption, and evaluation strategies, which were utilized in phases II and III. Data have been collected from 2015 to 2018 and analyzed. The results will be reported elsewhere.

**Conclusions:**

The IM protocol provided a rigorous framework for the development of a multicomponent evidence-based intervention to promote pulse-based foods in childcare centers.

**International Registered Report Identifier (IRRID):**

RR1-10.2196/22775

## Introduction

Consumption of plant-based foods has gained attention worldwide for many reasons, including health, environment, and animal welfare. Recognizing the health benefits of plant-based proteins [1-4], the recent release of Canada’s Food Guide (2019) and the Planetary Health Diet both emphasize consumption of these foods [5,6]. Pulses (dry beans, lentils, chickpeas, and peas) are excellent sources of plant-based proteins that can improve the quality of diets; additionally, these foods are rich in micronutrients and fiber and low in fat, and have a low glycemic index.

Despite the health benefits and the high production of pulses in Canada, their consumption is low among Canadian adults [7]. Studies have shown that the development of eating behaviors and food preferences begins during the early years (0-5 years of age) [8,9]. Although there is a paucity of data on children’s consumption of pulse-based foods at the national level, Mudryj et al [10] reported that only 8.2% of children residing in Manitoba consumed pulses or soy. Additionally, Jarman et al [11] noted that there is a need for improvement in the diets of preschool-age children, with 90% and 35% of preschool children consuming more than 20 g of candy and snacks (such as popcorn, pretzels, and cookies) and 100 g of sugar-sweetened beverages, respectively, each day.

Understandably, the home and family environments have an important influence on children’s eating behaviors. However, according to a survey released in 2019, about 52% of Canadian children under 6 years of age were enrolled in childcare centers [12]. Given that children typically eat at least two meals each day in childcare centers, these centers make an ideal venue to engage a large proportion of children for nutrition-related behavior change interventions. Engaging parents to reinforce such center-based nutrition interventions was found to be crucial in achieving the intended goal [13].

It has been argued that consistently exposing children to nutritious pulse-based foods at an early age may help to promote healthy dietary behaviors that can span into adulthood [14]. Implementing such a behavior change is a complex process that requires a systematic approach. The intervention mapping (IM) protocol provides a framework for guiding the design and implementation of multilevel nutrition education interventions. IM has been successfully used to create and promote a healthy diet in several nutrition behavior change interventions that target children aged 3 to 13 years, as well as their parents [15-20]. Interventions designed using such a structured framework to encourage pulse consumption are scarce. This paper describes the utilization of IM in the development of a multicomponent nutrition intervention aimed at promoting consumption of pulse-based foods among preschool children in childcare centers in Saskatchewan, Canada.

## Methods

### Overview

The study encompassed three phases. Phase I focused on the development of the Pulse Discovery Toolkit (PDTK) intervention (described in IM steps 1-6 listed below), phase II encompassed pilot testing, and phase III included intervention roll-out with minimal support from the research team. In total, one, two, and four childcare centers participated in phases I, II, and III, respectively. Community partners (childcare center directors, early childhood educators, cooks, and parents) were consulted at various points of the study.

### Intervention

The PDTK is a multicomponent nutrition education intervention designed using a stepwise IM approach [21] with two theoretical frameworks, namely the Piaget cognitive developmental theory and social cognitive theory (SCT). The six-step process that guided the developmental process is described below.

#### Step 1: Needs Assessment

The first step in the development process was a needs assessment and an analysis of the problem. This step was completed through stakeholder (educators, parents, researchers, and community partners involved in the field) consultation, informal conversations with childcare center staff, and a literature review.

Through the literature review, the consumption of pulse-based foods was explored to elicit additional information on barriers and determinants. Additionally, food-based interventions conducted in childcare settings were reviewed to identify gaps and successes that would be helpful for the development of this intervention. For the stakeholder consultation process, at the beginning of the study, an eight-member multidisciplinary team of researchers, graduate students, and community partners was established to conduct problem analysis, fine-tune the PDTK intervention, and participate in recipe selection and taste testing. Two early childhood educators (ECEs) and preschool children (n=23) from a pretest childcare center in Saskatoon, Saskatchewan, Canada, who were not involved in the pilot study or roll-out study, took part in the development of the PDTK intervention. This pretest site in Saskatoon was chosen owing to its proximity to the University of Saskatchewan campus and willingness to participate in the PDTK development process. Informal discussions were carried out with the pretest childcare center staff on findings obtained from the literature review. The literature review revealed that the emphasis of nutrition interventions targeting preschool children in childcare settings has been on the consumption of fruits and vegetables [22-25]. Furthermore, studies indicated that most effective interventions were multicomponent and involved children, parents, educators, and the food environment [26,27].

**Figure 1:**
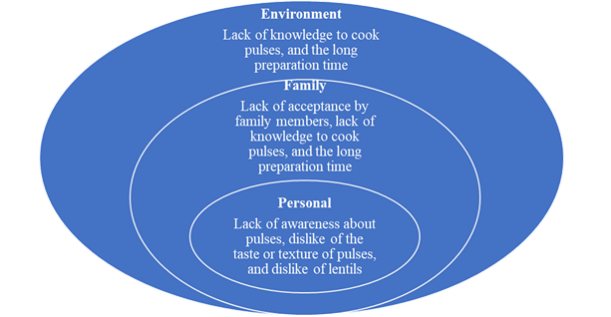
Individual, social, and environmental determinants of pulse consumption among preschool children based on literature review.

Determinants of pulse consumption at individual, social, and environmental levels identified from the literature [28,29] are presented in Figure 1. Very few studies in Canada have addressed these determinants. A childcare center intervention study conducted by Froehlich Chow et al [30] focused on increasing educators’ and cooks’ knowledge of pulses that increased their knowledge and utilization of pulse-based foods in these settings. 

Recipe books and websites were reviewed to create a matrix of child-friendly pulse-based recipes for lunch and snacks that could be adopted in the intervention using predefined criteria (Multimedia Appendix 1). The Kids Book of Lentils; Pulses; Cooking with Beans, Peas, Lentils and Chickpeas; Lentils for Every Season; The Best of Heart Smart Cooking; and Pulse Canada websites were among the resources reviewed for recipes [31,32]. In addition to pulse-based foods that could be prepared at childcare centers, commercially purchased products, such as puffs, lentil pasta, and biscuits, were also included in the matrix.

The criteria for recipe selection were developed by consulting different guidelines, but mainly focusing on the 2007 Eating Well with Canada’s Food Guide and Saskatchewan Ministry of Education’s Child Care Regulations [33,34]. Criteria considered during the selection of recipes were as follows: (1) capacity to offer 0.5 to 1 food guide serving, with at least two food groups for mixed dishes; (2) whether the food item could be eaten by children independently; (3) risk of choking; (4) availability of ingredients; (5) risk of allergy; (6) cost of ingredients; and (7) ease of preparation. This initial selection process generated a possible list of 30 pulse-based recipes (Multimedia Appendix 1).

**Figure 2:**
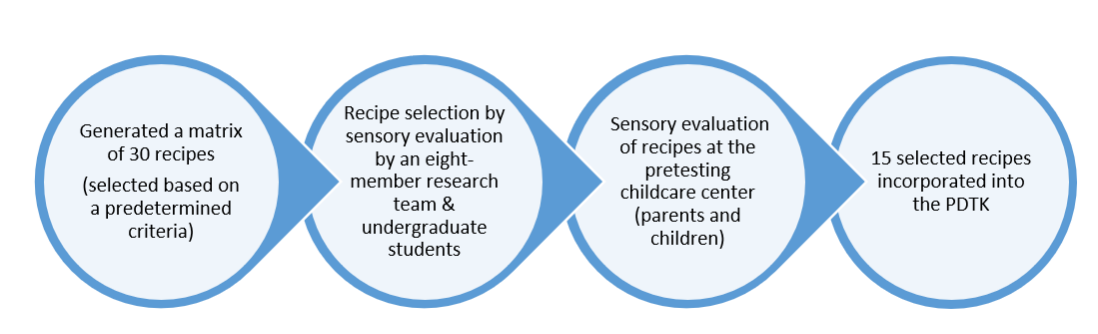
Step-by-step recipe selection process for integration into the Pulse Discovery Toolkit intervention.

The eight-member team, along with undergraduate nutrition students, preschool children, and their parents in the pretest center participated in sensory evaluation of the recipes at different stages of the PDTK development (Figure 2). Initially, the recipes were evaluated by the eight-member team for taste, appearance, and the likelihood of acceptance in a childcare center. This evaluation resulted in a list of 11 acceptable recipes that could be incorporated without any modifications into the PDTK, as well as a few recipes that would require further modification such as reducing strong flavors, increasing the proportion of pulses, and improving texture. After the recipe modification, students in an undergraduate nutrition class were invited to taste four of the recipes that needed modifications based on the team’s evaluation to see if they needed additional adjustments. Further sensory evaluation of selected recipes was then carried out with preschool children at the pretest center to determine their acceptability. A modified method previously used in kindergarten settings was employed for this assessment [35]. Groups of three to five children of similar ages were paired with one researcher. Researchers explained the procedure to the children, and samples of pulse-based foods were presented to them. They could ask for more portions if they wanted. The researchers observed and took detailed notes on whether the children ate all, some, or none of the portions or if they asked for more. The reports from each researcher were used to record how many children tasted the foods, and the results indicated that the majority of children were willing to taste the recipes and expressed their liking. In addition to gathering information on willingness to taste and liking of the pulse-based foods, this process also helped in shaping the final sensory evaluation procedure to be used for the pilot testing project. Parents and their children attending a parent-teacher consultation meeting at the pretesting site also provided an informal evaluation of two of the recipes (lentil smoothie and lentil pizza). Parents tasted the recipes and provided feedback verbally. Most parents indicated that they liked both recipes. Overall, this process served to identify 15 recipes that were incorporated into the PDTK.

#### Step 2: Specifying Program Objectives

Findings from the expert consultation and literature review in step 1 helped to shape the desired objectives of the PDTK intervention. The general program objective was to increase the consumption of pulse-based foods among preschool children in childcare settings. Considering the barriers and determinants related to consumption of pulses by children identified in step 1, the program objective was translated into performance objectives at individual (children), and social and environmental levels (parents, ECEs, and cooks). These are activities that the children, parents, ECEs, and cooks needed to complete to promote behavior change. Parents, ECEs, and cooks were identified as key players who could potentially influence children’s food choices [26,36] and were a focus when planning the intervention. The objectives that focused on children, parents, ECEs, and cooks to increase consumption of pulse-based foods among preschool children are presented in Textbox 1.

**Performance objectives of the Pulse Discovery Toolkit intervention at individual (children), and social and environmental levels (parents, early childhood educators, and cooks).**

Performance objectives at the individual level (preschool children)

- Name pulses
- Grow pulses
- Identify to which food group pulses belong
- Try pulse-based dishes
- Tell parents about pulses
- Ask for pulses in different settings
- Participate in the preparation of simple pulse-based dishes

Performance objectives at the social and environmental levels (parents, early childhood educators, and cooks)

- Teach children pulse-related concepts
- Prepare pulse-based dishes
- Incorporate pulse-based food in children’s menus

#### Step 3: Selecting Theory-Based Methods and Practical Strategies

Studies have shown that implementing a successful nutrition education intervention requires a theoretical component [37-39]. For the PDTK, two theoretical frameworks were selected to guide intervention development. The frameworks selected for this intervention were the SCT and Piaget cognitive development theory.

The most dominant and extensively used theory for implementing nutrition education programs for children is the SCT [40]. The SCT is known for its comprehensive approach, taking into consideration environmental, personal, and behavioral factors [41], which help to define intervention components [42]. This theory is often chosen because of its “emphasis on approaches that are important to youth, such as positive reinforcement” [42]. The SCT is also considered an effective framework for program development owing to interactions between individuals, their environment, and learning capacity [43]. The constructs of behavioral capability, observational learning, positive reinforcement, and environmental changes were used as guidelines in constructing the PDTK intervention components. Even though the SCT is used as the main theoretical framework, the developmental stage of preschool-aged children makes it difficult to apply all SCT constructs, such as self-evaluation. Recognizing this, the Piaget development theory was used to complement the SCT. The Piaget cognitive development theory offers specific explanations of a child’s cognitive development.

The Piaget cognitive development theory highlights play and self-discovery and provides direction in the preparation of age-specific education content. According to Piaget, there are four developmental stages of children, which progress in a linear fashion. The preoperational period, which is most applicable to PDTK development, focuses on children aged 2 to 7 years, is classified as the second stage of a child’s cognitive development, and involves the development of symbolic thought and consideration of the world through an egocentric perspective [44]. This stage of development is the most applicable to children during the preschool years, as at this stage, a child cannot use logic or combine ideas and only learns by copying the environment, discovering, questioning, classifying, socializing, and tangibly understanding concepts [45]. This copying of the environment, discovery, and questioning result in a schemata perception that influences children’s new experiences [46]. Hence, a new schema can be formed in children’s minds about new foods, such as pulses, if they are exposed to them through educational activities.

Taken together, both theories helped to create the project’s strategy to encourage children to adopt healthier diets and improve pulse consumption. For example, creating social and environmental changes through constant exposure to pulses and pulse-based dishes was expected to increase preferences for and consumption of pulse-based dishes. Previous studies have shown that repeated taste exposure can influence liking and willingness to consume new foods [47,48]. Subsequently, a matrix of objectives matching the performance objectives defined in step 2 was developed based on the constructs of the SCT while taking children’s cognitive development into consideration (Table 1 and Table 2). In addition to the SCT and Piaget cognitive development theory, other theory-based methods were considered and implemented where applicable. These included active learning, sensory learning, and imagery. For example, children were encouraged to actively participate in creating their own pulse-based wrap, taste testing pulse-based buffets, growing pulses in a pot, naming pulses on a picture, and creating crafts using pulses.

**Table 1:** A matrix of performance objectives for preschool children in the Pulse Discovery Toolkit intervention.

Performance objectives	Knowledge	Skills	Self-efficacy
1. Name pulses	Describe pulses	Identify different types of pulses	Confidence in pulse identification
2. Grow pulses	Describe pulse gardening	Develop gardening skills	Confidence to participate in pulse gardening
3. Identify to which food group pulses belong	Describe the benefits of pulses	N/A^a^	Confidence in pulse identification
4. Try pulse-based dishes	Describe the taste of pulse-based dishes	Describe the taste of pulse-based dishes	Confidence in tasting pulse-based dishes
5. Tell parents about pulses	Describe pulses to parents	Describe pulses to parents	Confidence to discuss about pulses
6. Ask for pulses in different settings	Identify pulses in a variety of places	Practice asking for pulses in a variety of settings	Confidence of pulse selection in a variety of settings
7. Participate in the preparation of simple pulse-based meals	Describe how to prepare pulse-based dishes	Practice food preparation skills	Confidence to participate in preparing simple pulse-based meals

^a^N/A: not applicable.

**Table 2:** Matrix of performance objectives for parents, early childhood educators, and cooks in the Pulse Discovery Toolkit intervention.

Performance objectives	Knowledge	Skills	Preference
1. Teach children pulse-related concepts	Describe pulses	Demonstrate an ability to teach pulse-related concepts	Teaching children about pulses will mediate pulse-based food intake
2. Prepare pulse-based dishes	Discuss cooking pulses	Demonstrate an ability to cook a variety of pulse-based dishes	Providing pulse-based dishes will help children develop a preference for pulse-based foods
3. Incorporate pulse-based foods in children’s menu	N/A^a^	Demonstrate an ability to plan a menu that incorporates pulse-based dishes	Incorporating pulse-based foods into center menus will expose children to different dishes containing pulses

^a^N/A: not applicable.

#### Step 4. Designing and Organizing the Program

During the fourth step, information obtained in the previous steps was organized into program components. The components of PDTK were designed to target the individual and environmental determinants identified in the previous step of IM. For example, the food service guide, which included a guide to cooking pulses in a childcare center, recipes, and shopping tips, could enhance the environmental conditions and reinforce pulse-related knowledge, thereby impacting purchase and consumption of pulses. The objectives pertaining to the behavior of parents and guardians were facilitated through the development of a newsletter as a mechanism to support their healthy eating behavior. The components of the PDTK encompassed the following: (1) a 12-week lesson plan, (2) a food service guide for incorporating pulses in the childcare center’s menu with an example of a 4-week cycle menu, (3) pulse recipes, and (4) four parent newsletters, which are described in more detail below.

##### Lesson Plan

**Figure 3:**
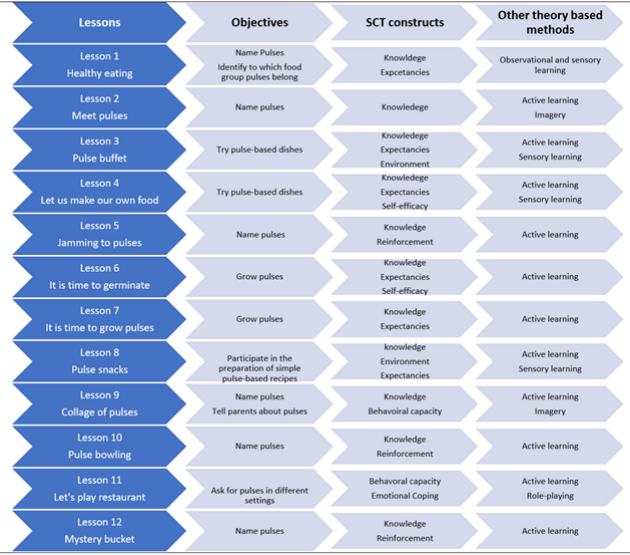
Matching the lesson plan activities of the Pulse Discovery Toolkit intervention with the social cognitive theory and other theory-based methods.

The lessons were designed to actively engage children in learning about healthy food choices with a focus on pulses. Though the emphasis was on pulses, the lessons targeted multiple health-related behaviors and domains of wellness. For example, physical health through nutrition and physical activity, mental wellness (self-efficacy and knowledge), and social wellness (problem solving and interacting with others during the lessons). The lesson plans consisted of child-friendly activities and learning objectives. The developed materials, including visual messages, pictures, handouts, worksheets, and exercises, were designed to increase familiarity to pulses. Practical strategies using the objectives defined in step 2 and the selected theories in step 3 were developed (Figure 3).

##### Food Service Guide

The menu was designed to complement the nutrition education resource with child-tested meals and snacks. To achieve lasting impact, it was planned that students would receive 15 to 20 exposures to pulses through lunches, snacks, and classroom sensory sessions during the 12-week intervention. Taken together, this resource was expected to help address young children’s reluctance to try new foods by providing different ideas to promote multiple exposures to pulse-based food products.

##### Parent Newsletter

Paper-based newsletters were sent home to the children’s families as a mechanism to introduce and facilitate pulse use in households (Multimedia Appendix 2). The newsletters included information about the project, the weekly lessons taught to the children, and pulse-based intervention recipes served in the childcare center. The first newsletter was sent to parents at the beginning of the project, while the remaining three were sent monthly.

#### Step 5: Specifying Adoption and Implementation Plans

This step focused on strategies to promote adoption of the PDTK and the development of an implementation plan. The adoption and implementation strategies included active involvement of primary stakeholders, such as ECEs, cooks, and directors of the childcare facilities. To ensure adoption, each lesson plan and its activities were evaluated for content, flow, and age-appropriateness during the pilot testing of the study. The pilot-testing process would ensure program component evaluation by the major stakeholders.

For implementation and pilot testing, the childcare center was provided with a PDTK manual that incorporated the program components. Additionally, the staff received training in implementing the program. Trained graduate students and ECEs provided the 12 PDTK lessons. The pulse-based foods served for lunch were mainly prepared by the cooks in the childcare center facilities. For pilot-testing, graduate students assisted in the preparation of pulse-based foods used for sensory evaluation. Overall, researchers provided minimal support during the roll-out compared with pilot testing.

#### Step 6: Evaluation Planning

In this final step, a plan to evaluate the feasibility and impact of the PDTK program was developed. A multimethod approach with both quantitative and qualitative data collection tools was designed. The quantitative approaches to evaluate feasibility and effectiveness included (1) sensory evaluation, (2) plate waste assessment, and (3) pre-post knowledge testing regarding pulses among children. For qualitative data collection, one-on-one semistructured interview guides were developed to elicit detailed information regarding the PDTK intervention from the ECEs and cooks. The ECEs in the pilot testing childcare center were asked to provide qualitative feedback through a predeveloped weekly lesson plan evaluation form. The lesson plan evaluation carried out in phase II pilot testing provided detailed feedback that was used to revise the PDTK before the roll-out in phase III.

##### Parent Sociodemographic Questionnaire

A 21-item questionnaire was developed using Statistics Canada’s Community Health Survey Questionnaire and the Daily Lentils Study [29]. Questions in the parent’s questionnaire included, for example, how often their child consumed pulses at home and parents’ highest level of education and household income.

##### Knowledge Assessment Questionnaire

Pretest and posttest assessments of children’s pulse knowledge were planned for each intervention site. A pictorial data collection questionnaire was developed, adapted, and pretested for this purpose. This type of pictorial tool has been previously validated and used with children [49,50]. The tool consisted of five questions with various images representing different varieties of pulses. The knowledge test was organized into different pulse categories, and for each category, children were asked to identify or to indicate if they recognized the types of pulses presented. Samples of relevant pulses were also presented to each child during the questioning to assist with this process.

##### Sensory Evaluation

Sensory evaluation of the pulse-based foods in the PDTK was conducted using a three-point facial hedonic scale with ratings of “Yummy,” “Yucky,” and “Ok” [51]. Evaluations were carried out by graduate students familiar with sensory evaluation techniques. The sensory protocol was adapted from a study conducted by Guthrie et al [52]. Following an introduction of the procedure to participant children in groups, each child’s understanding of the scale was assessed on an individual basis using samples of fruits (eg, pineapple, strawberries, honeydew melon, and cantaloupe). Each child was asked to pick the fruit that he or she thought was “Yummy” and asked to select the corresponding face representing the taste of the fruit. In between tasting and rating each sample, the children were asked to rinse their mouths with water. The training was completed when the children were able to understand each facial category properly.

Sensory evaluation of the pulse-based foods by the children was conducted as part of the lesson plan, and during the snack and lunch periods. As part of the lessons, the evaluation was carried out on the following three occasions: during the lessons “Pulse Buffet” (lesson 3), “Let’s Make Our Own Food” (lesson 4), and “Pulse Snacks” (lesson 8). Selected recipes were evaluated at two different time points to compare changes in liking after repeated exposures. Preference testing was also conducted on the selected spreads to determine children’s liking of the recipes. The procedure included presenting two different samples of spreads (red bean spread and green split pea spread) to the children and asking them to identify which one of the samples they preferred.

##### Plate Waste

**Figure 4:**
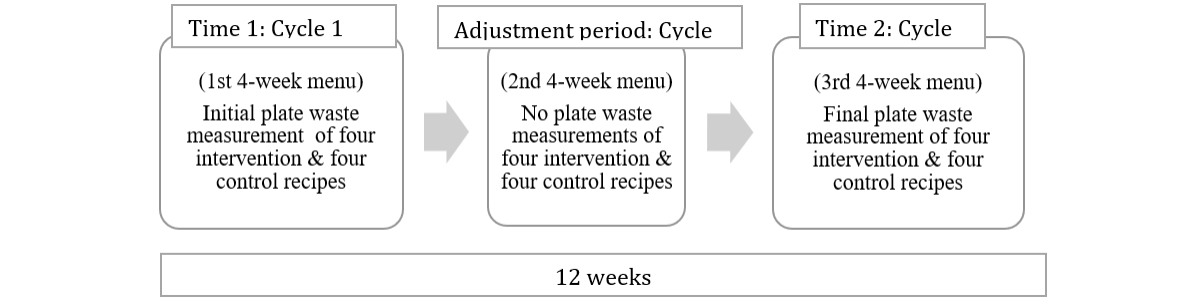
The plate waste measurement cycle in the Pulse Discovery Toolkit intervention.

Plate waste measurements of the intervention and control recipes were taken twice during the 12-week intervention as shown in Figure 4. Using each childcare center’s 4-week cycle menu, four intervention pulse-based dishes (chickpea spread, three bean quesadillas, lentil pizza, and chicken stir fry) were randomly assigned to weekdays in the menu. The control recipes were chosen from each childcare center’s regular menu and designed to fall on the same day of the week as the intervention recipe during the 4-week cycle menu. The foods served to the children were weighed and photographed before and after each meal. No plate waste measurements were taken during the 4-week adjustment period of the second cycle (cycle 2); however, the intervention recipes were incorporated into the menu to increase familiarity with the recipes.

Information on plate waste was captured using a digital photography weighted plate waste app validated previously [53]. Briefly, a photographed reference of the selected dishes was taken prior to each meal service to provide a pictorial representation (sample) of the food served and eaten by each child. Refuse or discarded food items were also photographed and weighed on a digital scale (Salter Magic Display Electronic Scale 10B55BKEF, Springfield Instruments). To ensure consistency, the same types of plates and utensils were used each day during the data collection period. The amount of food consumed was obtained by subtracting the amount of plate waste from the amount of food served.

##### Comparison of the Nutrient Composition of the Intervention and Control Recipes

Esha Food Processor Nutritional Analysis Software (version 10.10.00) was used to code and compute the nutrient breakdown for all the intervention and control recipes. The nutrient composition of control recipes (four recipes from each center) and four intervention recipes were compared. Nutrients compared between recipes included kilocalories, protein, fiber, fat, saturated fat, calcium, potassium, sodium, and iron content. In addition, the number of food groups provided by a reference serving for each recipe was determined using the Eating Well with Canada’s Food Guide [33]. To calculate the number of servings within each food group, the amount consumed (grams or milliliters) was divided by the value from Canada’s Food Guide’s reference for one serving of that food group. For example, if a child consumed 15 g of crackers and the reference for crackers is 30 g per serving, the child consumed a 0.5 serving of grain products. If the food item did not fall into one of Canada’s Food Guide food groups, it was assumed to belong to the “other” category.

##### Lesson Plan Evaluation

Process evaluations were administered through a weekly lesson plan evaluation. The lesson plan evaluation forms included questions about the feasibility of the activities and acceptability (ie, if the children were engaged in each lesson), as well as requested suggestions from ECEs about how to improve each lesson. Questions for the lesson plan evaluation were adapted from a previous study conducted by Sharma et al [22] involving a pilot study of preschool-based healthy nutrition and physical activity programs.

##### ECE, Director, and Cook Interview

A semistructured postintervention interview explored the level of satisfaction with the PDTK program. Questions were adapted from the report by Sharma et al [54]. Interview questions explored topics such as nutrition concepts, children’s level of engagement throughout each lesson, and suggestions to improve the nutrition education resource. The cooks’ and directors’ interview guides were designed to assess the perceived benefits and barriers of cooking pulses and the feasibility of cooking pulse-based dishes. In addition, the questions were designed to generate information regarding PDTK recipes the cooks least enjoyed and most enjoyed cooking. Their perceptions of whether the children liked the pulse-based dishes and whether these dishes should be incorporated into the menus of the childcare centers were also discussed during interviews.

#### Data Analysis

Quantitative data were analyzed using SPSS version 24 (IBM Corp) and SAS version 9.4 (SAS Institute Inc). SPSS was used to generate descriptive statistics and for paired comparisons. Chi-square and McNemar tests were used for paired nominal data to compare the preindividual and postindividual pulse knowledge scores. Data analysis techniques were adapted from a study conducted by Sigman-Grant et al [55]. A paired sample *t* test was also conducted on spreads (green split pea vs red bean) to determine if there was any difference between sensory acceptance at the beginning and the end of the intervention. SAS was used to generate *t* test and generalized estimating equation model results for plate waste measurements. The consumption proportions were calculated for each of the intervention and control recipes using the following formula: consumption proportion = sum of the amount of all intervention (or control) foods a specific child eats / sum of the amount of intervention (or control) foods given to that specific child. Using aggregate data, an independent *t* test was performed to compare the mean consumption and the mean consumption proportions of both the intervention and control recipes. Results were considered statistically significant if a *P* value of <.05 was obtained.

For qualitative data analysis, the audio recordings of the semistructured interviews were transcribed verbatim. The transcripts were then sent to the interviewees for verification. The verified transcripts were then analyzed in three phases. The first phase involved an initial review of the transcript for common concepts and themes. The process of analysis consisted of reading each individual response to each question several times and highlighting the major ideas that emerged from the transcript. The second phase of analysis consisted of an auditing process, where concepts or themes were reviewed by an independent researcher as a means of verification, to determine if there was common consensus or disagreement with the themes identified in the initial analytical process. After the verification process, the third phase involved rearranging quotes into the selected thematic categories. Subsequently, comparisons were made between categories to describe the findings [56].

#### Ethical Consideration

Ethical approval was obtained from the University of Saskatchewan Behavioral Research Ethics Board. Prior to data collection, consent and verbal assent were obtained from parents and children, respectively.

## Results

**Figure 5:**
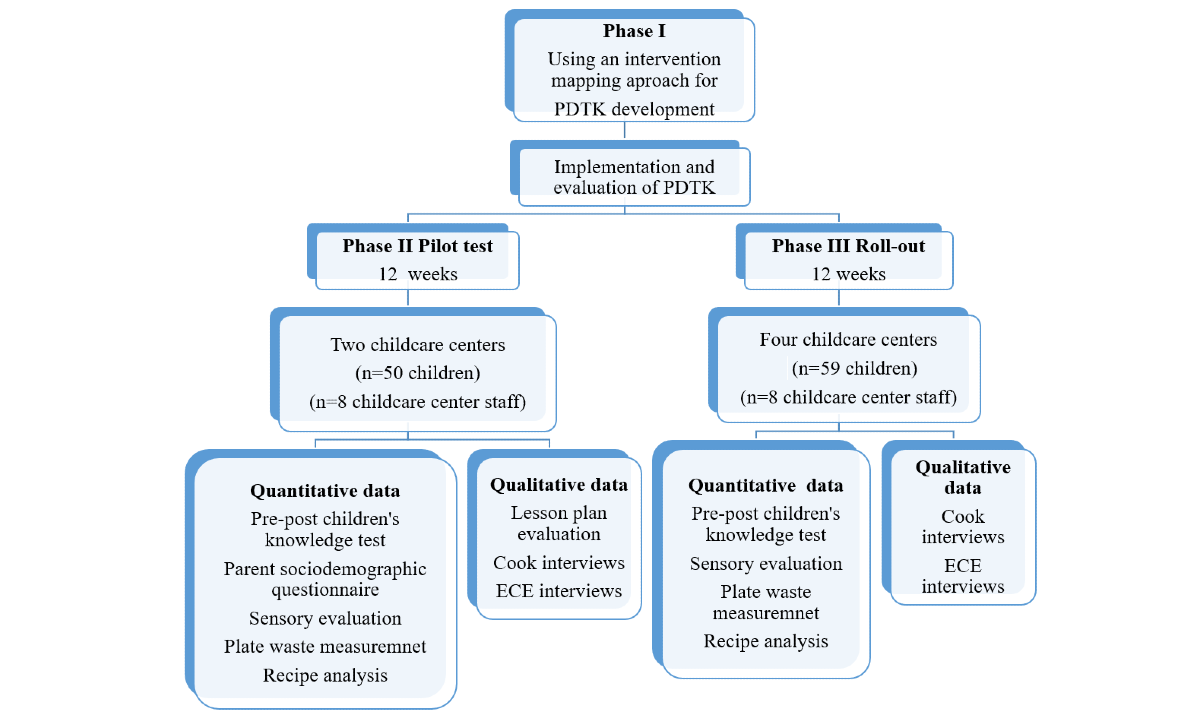
Flow chart showing the development of the Pulse Discovery Toolkit (PDTK) intervention, its implementation, and the evaluation process. ECE: early childhood educator.

Data have been collected from 140 participants from 2015 to 2018 and analyzed. The results will be reported elsewhere. Figure 5 presents the types of data collected at different phases of the study.

## Discussion

### Overview

Studies have widely documented the health benefits related to the consumption of plant proteins, such as lower risk of cardiovascular disease, lower blood pressure, and improved diabetes management [1,2,4]. However, a recent study underlines the presence of knowledge gaps about plant protein food consumption and the need for food literacy to enhance health [57]. It is reasonable that the introduction of the concept of plant proteins should start at an early age for lasting impact. Pulses are locally produced plant proteins that are poorly consumed in Canada. The PDTK was designed to address the knowledge gap about pulses and provide repeated exposure to pulse-based foods, cooking tips, child-friendly pulse-based recipes, and information for parents though newsletters.

This protocol describes the use of IM in the development of a multicomponent nutrition intervention to promote the consumption of pulse-based foods among preschool children attending childcare centers. Through needs assessment, consumption of pulse-based foods was identified as the behavior targeted for change. This initial step in IM ensured an in-depth understanding of the problem and helped to tailor program components to the findings of the needs assessment in later stages of planning. An important contribution of the IM process was the ability to systematically identify gaps, successes, and determinants before designing program components.

In step 2, the IM process facilitated the selection of determinants and assisted in narrowing the general program objective to objectives changeable at individual, social, and environmental levels (parents, ECEs, and cooks). Changes at the policy level were not assessed as this was beyond the scope of the project. This step facilitated the selection of the most important and modifiable behavioral determinants to ensure a change in behavior. The performance objectives were not only limited to nutrition behaviors, and multiple domains of health were targeted to provide comprehensive intervention. Fernandez et al [58] described this as a critical step in planning as it distinguishes between behaviors and their determinants, and helps establish clear performance objectives.

In the third step, the application of IM facilitated the systematic selection of theories. The available evidence indicates that most theory-based nutrition-related interventions are effective [27]. This step provides a practical decision-making process during the development of the intervention, which is beneficial to health education program planners [21]. Step 4 focused on creating program components and was instrumental in linking the program components to already identified behaviors and determinants.

IM further assisted in capturing and incorporating the opinions of stakeholders during the development process, hence allowing program planners to plan for adoption and implementation in step 5. The support of stakeholders is crucial for better adoption and scale-up [58]. As discussed in the study by Froehlich Chow et al [30], once ECEs noted that children tasted and liked the pulse-based foods, they were motivated to serve them more often. In the final IM step, a comprehensive evaluation plan was developed.

### Limitations

One of the drawbacks of the IM process was that it was time-consuming and complex. Similar limitations have been documented in interventions using IM on related topics [15,59]. Steps 1 to 3, which particularly defined the performance objectives and determinants of pulse consumption and matching with the theoretical framework, required a substantial amount of time. In addition, the process requires going back and forth to ensure all aspects are correctly addressed. Though the SCT has been mostly used to explain the determinants of fruit and vegetable consumption, the selection of the most effective SCT constructs was limited owing to the lack of interventions utilizing the SCT for pulse-based foods in preschool children. The other limitation associated with the SCT, which was owing to the age of the children, was inability to apply all of the SCT constructs. For example, the self-control SCT construct was difficult to design and implement with preschool children. Another limitation in this study is related to parents’ engagement, although attempts have been made to engage parents through newsletters. Future research should look for innovative ways to engage parents and increase their involvement in studies in childcare settings.

### Conclusion

The six-step IM ensured the development of an evidence-based nutrition intervention. The initial step in the IM process confirmed the need for an intervention targeting pulse-based food consumption. The IM process facilitated the identification of determinants of dietary habits focused on pulse-based food consumption, allowed the use of multiple theoretical frameworks, and assisted the matching of program objectives with the theoretical framework and lesson plan. Although the process was time-consuming, it provided more clarity to various components of the intervention, thereby increasing the intervention’s effectiveness potential. To the best of our knowledge, this study is unique as it is the first of its kind to introduce pulse-based foods to young children aged 3 to 5 years through the application of a systematic theory-based IM approach in a childcare setting. Results generated from the evaluation component of this study will guide further improvements of the PDTK intervention.

## Appendix

Multimedia Appendix 1 Matrix of pulse-based recipes and selection.

Multimedia Appendix 2 Parents’ newsletter.

## Abbreviations

ECE: early childhood educator
IM: intervention mapping
PDTK: Pulse Discovery Toolkit
SCT: social cognitive theory
