# Supplementary material for: A Nutrition Intervention to Promote the Consumption of Pulse-Based Foods in Childcare Centers: Protocol for a Multimethod Study
Source: JMIR Res Protoc. 2020 Dec 24;9(12):e22775. doi: 10.2196/22775 (PMC7790610; doi:10.2196/22775)
Supplement: Multimedia Appendix 1 [file resprot_v9i12e22775_app1.docx]

**Multimedia Appendix 1- Matrix of pulse-based recipes and selection.**

**A Matrix of Pulse-Based foods**

1. Lentil pizza

2. Granola bar (lentil)

3. Lovely lentil smoothie

4. Bean cookie

5. Lentil cookie

6. Stir fry chickpea chicken & vegetable

7. Red pea’s soup

8. Three bean quesadillas

9. Black bean antojito

10. Split peas soup with dill

11. Spinach eye ball

12. Smoky sloppy joes

13. Mashed potato with bean

14. Blueberry lentil muffin

15. Chickpea brownie

16. Lentil lasagna

17. Chickpea spread

18. Refried beans

19. Spaghetti bolognese

20. Cranberry orange muffins

21. Chickpea spread

22. Lentil spread

23. Green pea spread

24. Red bean spread

25. Bean Tacos

26. Garlic pesto biscuits

27. Hot chocolate biscuits

28. Lentil Pasta

29. Chickpea & Lentil puffs (Chili)

30. Chickpea & Lentil puffs (Lemon)

**PDTK Menu Items Final List**

1. Granola bar (lentil)

2. Cranberry orange bean muffin

3. Split pea soup with dill

4. Bean cookie

5. Stir fry chicken with chickpeas

6. Spaghetti bolognese (lentil)

7. Mashed potato with white bean

8. Three bean quesadillas

9. Lentil pizza

10. Refried bean

11. Lovely lentil smoothie

12. Green pea spread

13. Red bean spread.

14. Chickpea spread

15. Lentil spread

**Final selection via sensory evaluation of recipes by research team, undergraduates, pre-school children and parents**
