# Supplementary material for: A Nutrition Intervention to Promote the Consumption of Pulse-Based Foods in Childcare Centers: Protocol for a Multimethod Study
Source: JMIR Res Protoc. 2020 Dec 24;9(12):e22775. doi: 10.2196/22775 (PMC7790610; doi:10.2196/22775)

**Multimedia Appendix 2: Parents’ newsletter 1.**

**The next few weeks at a glance…**

The first few weeks of lessons are dedicated towards familiarizing your child with Canada’s Food Guide and the importance of eating a variety of foods. Your child will have lessons on nutrients they need to eat every day and the four food groups; also, they will learn where pulses are grown in Canada! We will introduce all the pulses through writing, creating a food rainbow, and colouring exercises. During the third lesson we will introduce the taste of pulses through a story of the three bears! The children will be introduced to a buffet of pulses, and we will ask your child to taste and give us feedback on the pulse based dishes. Ask your child about what they are learning and see how much they remember!

**Incorporating Local Pulses into the Menus of Childcare Centers in Saskatchewan**

**About the project:**

Starting Young is a study that uses a program design called Pulse Discovery Tool Kit. This includes the instructions, recipes, and other details about the study your child’s caretakers will use to complete this study. The Tool Kit is a multicomponent nutrition intervention designed to promote healthy eating habits in childcare centers. The Tool Kit is designed to specifically encourage incorporating pulse-based foods into the diets of pre-school children. These new foods will be introduced through fun and engaging activities and lesson plans held each week. Some of these lessons include doing art, reading, doing puzzles, recipe activities, and more!

The Pulse Discovery Tool also includes a 4-week cycle menu that allows your child to explore

different types of pulse-based products. We do not want you to feel left out though so we will provide you with 4 newsletters that includes recipes and information about what your child has been up to in our classes! These recipes are simple and great to include in your weekly meal plans at home!

Starting Young

**Did you know?**

Positive food experiences early in a child’s life helps promote optimal health, growth and development. These

experiences also contribute to the

prevention of chronic disease later in life.

**What are pulses?**

Pulses are a dried seed from a plant. Pulses include dried peas, beans, lentils, and chickpeas.

Pulses are high in protein, and low in fat. Pulses also have high levels of fiber and iron!


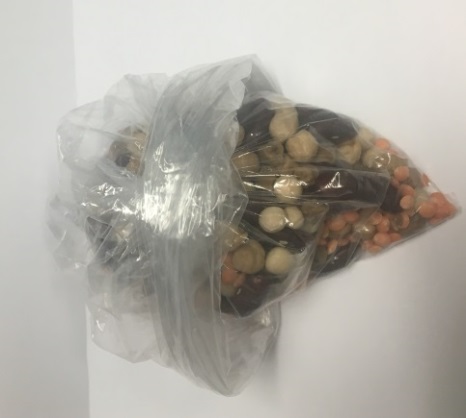

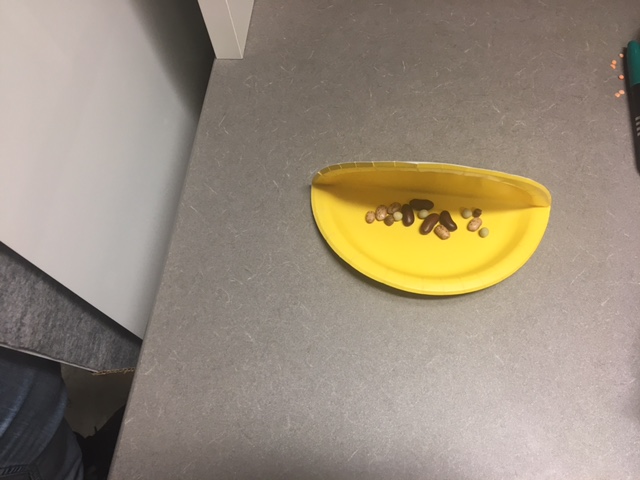

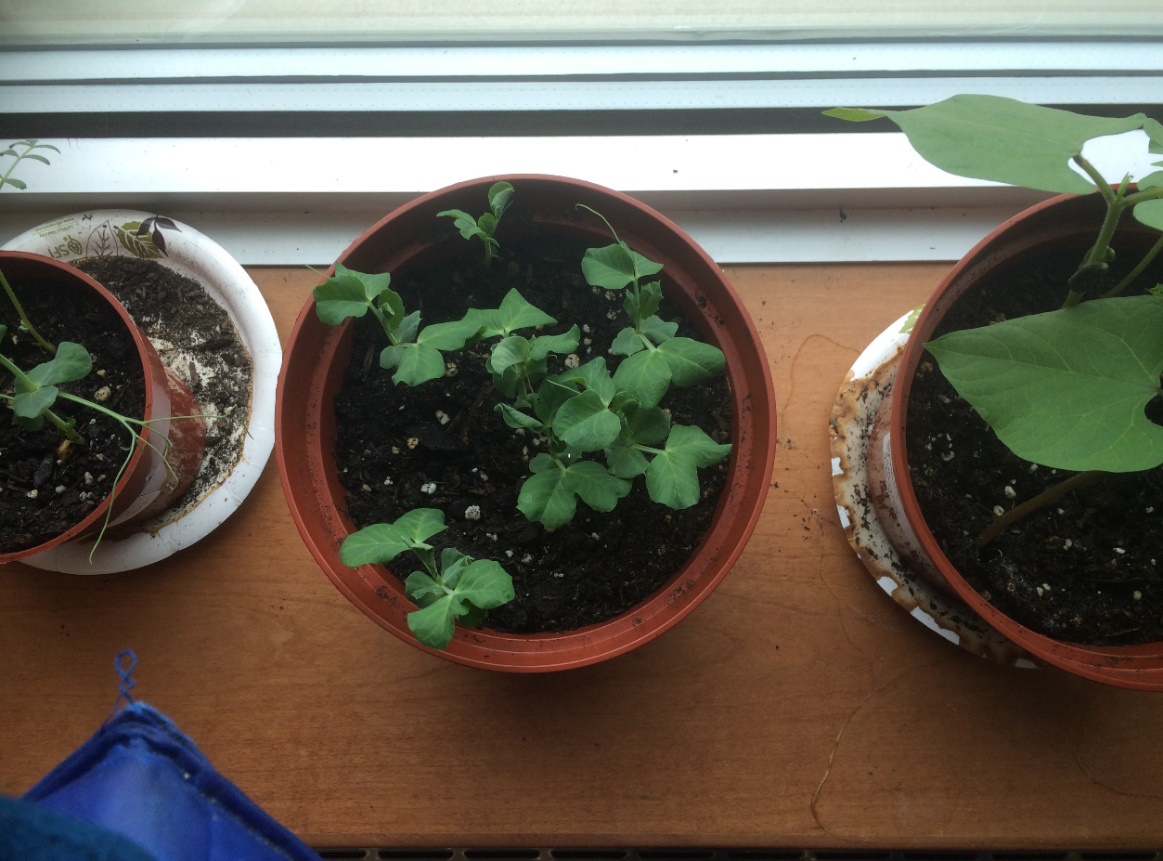

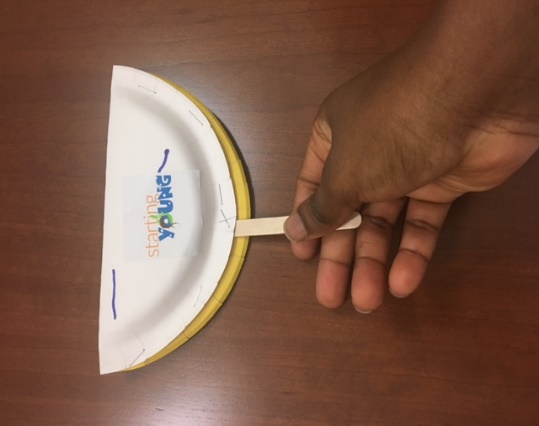

Supplement: Multimedia Appendix 2 [file resprot_v9i12e22775_app2.docx]
